# Association Study Between *NOTCH2* Gene and Idiopathic Central Precocious Puberty in Korean Girls

**DOI:** 10.3390/ijms27156686

**Published:** 2026-07-27

**Authors:** Young Suk Shim, Kyung Hee Kim, Min Hyung Cho, Jae Hyuk Oh, Hae Sang Lee

**Affiliations:** Department of Pediatrics, Ajou University School of Medicine, Suwon 164999, Republic of Korea; royjays@aumc.ac.kr (Y.S.S.); heeya09@aumc.ac.kr (K.H.K.); chomh89@ajou.ac.kr (M.H.C.); ssacktung@aumc.ac.kr (J.H.O.)

**Keywords:** central precocious puberty, *NOTCH2*, genetic association study

## Abstract

Identifying genetic contributors to idiopathic central precocious puberty (CPP) beyond established monogenic causes remains a research priority, as most cases lack a resolved genetic basis. We performed comprehensive Sanger sequencing of all 34 coding exons of *NOTCH2* in 100 Korean girls with idiopathic CPP and 100 age-matched healthy female controls. Among 55 identified sequence variants, including 11 missense variants, the primary finding was a statistically significant frequency difference for the *NOTCH2* heterodimerization domain C (HD-C) subdomain variant p.Ile1689Phe: this variant was absent from all 200 CPP alleles while present in 6.5% of control alleles (Fisher’s exact *p* = 0.0002), raising the hypothesis of a candidate protective association that requires independent replication before any causal inference can be drawn. Three additional missense variants, including p.Thr235Ser, p.Ala1361Thr, and the novel ankyrin (ANK) domain variant p.Ala1992Val, were identified exclusively in CPP patients as singletons; however, no pathogenic interpretation is warranted from these observations alone. This preliminary study reports a significant *NOTCH2* variant frequency difference and raises the hypothesis that *NOTCH2* coding variants may contribute to pubertal timing modulation within the Notch signaling pathway; independent replication and functional validation are required before causal conclusions can be established.

## 1. Introduction

Central precocious puberty (CPP) is the premature activation of the hypothalamic–pituitary–gonadal (HPG) axis before age 8 in girls and 9 in boys [1]. It leads to early secondary sexual characteristics and accelerated skeletal maturation, which can significantly reduce adult height if untreated [2]. CPP occurs predominantly in girls, with a female-to-male ratio of approximately 10:1, and the vast majority of affected girls are classified as idiopathic, meaning that no structural brain lesion or syndromic etiology is identified on clinical and radiological evaluation [3]. Beyond its impact on linear growth and skeletal development, CPP carries important psychosocial consequences, and it has been associated with increased long-term risks of reproductive and metabolic disorders, underscoring the clinical imperative to understand its underlying mechanisms [4].

The onset of puberty is governed by a complex neuroendocrine network, primarily driven by the pulsatile secretion of gonadotropin-releasing hormone (GnRH). This process is regulated by a delicate balance between excitatory inputs, such as the kisspeptin system, and inhibitory brakes, most notably represented by Makorin ring finger protein 3 (*MKRN3*) [5,6]. Disruption of this excitatory–inhibitory balance by pathogenic variants can lead to CPP. To date, four principal monogenic causes of CPP have been identified: activating mutations in Kisspeptin 1 (*KISS1*) and Kisspeptin 1 receptor (*KISS1R*), and loss-of-function mutations in the imprinted genes *MKRN3* and Delta-like non-canonical Notch ligand 1 (*DLK1*) [7].

Despite these advances, identifiable monogenic defects account for only a minority of idiopathic CPP cases. Recent evidence suggests a more complex genetic architecture, potentially involving polygenic or oligogenic contributions [8,9]. This has shifted research focus toward candidate pathways involved in GnRH neuronal development and gonadotropin secretion, including the evolutionarily conserved Notch signaling system [10,11]. The discovery of *DLK1*, a non-canonical Notch ligand, as a causative gene for CPP has already established a direct link between Notch pathway dysregulation and premature pubertal activation [12]. Among the Notch paralogs, Neurogenic locus notch homolog protein 2 (NOTCH2) is of particular interest. While experimental models implicate NOTCH2 in pituitary gonadotrope differentiation and GnRH neuronal ontogeny, a whole-exome sequencing study of Korean families with familial CPP identified a rare *NOTCH2* missense variant co-segregating with CPP in affected siblings [13]. Notably, this variant was present in isolation in an unaffected parent, and its pathogenic effect was proposed to arise through a digenic mechanism involving a concurrent *HERC2* variant with both genes operating within the Notch signaling axis—suggesting that synergistic perturbation of the pathway may lower the threshold for premature HPG axis activation [13]. Despite this preliminary evidence, a systematic mutational analysis of *NOTCH2* across its full coding sequence in a large-scale case–control cohort is currently lacking [14]. Consequently, the full spectrum of *NOTCH2* coding variants and their specific association with CPP susceptibility remain uncharacterized. In this study, we aimed to investigate the prevalence and clinical significance of *NOTCH2* variants in a cohort of patients with CPP.

We focused on *NOTCH2* as a candidate gene based on its direct role in pituitary gonadotrope differentiation and its position downstream of the established CPP gene *DLK1* within the Notch signaling cascade. To confirm that established monogenic causes of CPP were not present in the study population, all enrolled patients underwent pre-screening for pathogenic variants in the known CPP-associated genes *MKRN3*, *DLK1*, *KISS1*, and *KISS1R*; no causative variants were identified in any patient, thereby strengthening the rationale for investigating *NOTCH2* as an additional candidate locus. In the present study, we performed comprehensive Sanger sequencing of all 34 coding exons and flanking intronic regions of *NOTCH2* in 100 Korean girls with idiopathic CPP and 100 healthy female controls with a normal age of menarche to characterize the landscape of *NOTCH2* coding variants and evaluate their associations with CPP. This candidate-gene approach was designed to identify both risk-conferring and potentially protective variants across the functional domains of the NOTCH2 receptor, thereby providing insight into the role of this signaling molecule in the regulation of pubertal timing in the Korean pediatric population.

## 2. Results

### 2.1. Clinical Characteristics of CPP Patients

Clinical and hormonal characteristics are summarized in Table 1. Of the 100 enrolled patients, the mean age at CPP diagnosis was 6.75 ± 0.47 years. Eighty-eight girls (88%) were classified as Tanner breast stage II at the time of diagnosis. Mean height and weight were 123.43 ± 4.92 cm (standard deviation score, SDS: 0.91 ± 0.93) and 26.12 ± 4.50 kg (SDS: 0.79 ± 1.00), respectively. The mean bone age was 8.83 ± 0.94 years, corresponding to a bone age advancement (BA–CA) of 2.05 ± 0.81 years. Predicted adult height (PAH) was markedly reduced at 150.43 ± 6.43 cm (SDS: −2.18 ± 1.41). GnRH stimulation testing demonstrated a mean peak LH of 8.05 ± 4.45 IU/L and peak FSH of 16.70 ± 5.44 IU/L, both consistent with centrally driven precocious puberty. On the other hand, the clinical and anthropometric characteristics of the 100 control subjects are summarized in Table 2. The mean age at enrollment was 13.91 ± 1.03 years, and the mean age at menarche was 12.51 ± 0.63 years, confirming normal pubertal timing and excluding central precocious puberty in all controls. Mean height was 157.52 ± 5.38 cm (SDS: 0.04 ± 0.95), mean weight was 47.69 ± 6.95 kg (SDS: −0.33 ± 0.88), and mean BMI was 19.20 ± 2.50 kg/m^2^ (SDS: −0.44 ± 0.95), all within age-appropriate normal ranges based on the 2017 Korean National Growth Charts [15]. Mean target height calculated from parental heights (father: 172.85 ± 5.40 cm; mother: 160.49 ± 4.40 cm) was 160.17 ± 3.65 cm (SDS: −0.17 ± 0.68). Mean bone age was 14.69 ± 1.44 years, consistent with chronological age and normal pubertal progression.

### 2.2. Variant Identification and Overall Distribution

Genotype distributions in all subjects conformed to Hardy–Weinberg equilibrium (*p* > 0.05). Direct Sanger sequencing of all 34 coding exons and flanking intronic regions of *NOTCH2* (transcript reference: NM_024408.6) identified a total of 55 sequence variants: 11 missense variants, 13 synonymous variants, 21 intronic variants, 1 insertion-deletion (INDEL), and 9 5′ untranslated region (5′UTR) variants comprising 7 single nucleotide variants (SNVs) and 2 INDELs. All variants, including singleton and novel variants, were confirmed by independent bidirectional re-sequencing from a fresh aliquot of the original genomic DNA extract. All detected variants were present in the heterozygous state. No homozygous alternate genotypes or high-impact variants (frameshift or nonsense) were identified in either group. Allele frequencies for all missense variants and representative synonymous variants are presented in Table 3.

### 2.3. Missense Variants: Distribution Across Functional Domains

Among the 11 missense variants identified (Table 3, M1–M11), three were detected exclusively in CPP patients and absent in all 100 controls: p.Thr235Ser (Exon 4; EGF-like repeat 6; rs200464440), p.Ala1361Thr (Exon 25; cbEGF-like repeat 35; rs138567855), and p.Ala1992Val (Exon 33; ankyrin repeat (ANK) domain; novel). The variant p.Ala1992Val has not been previously registered in dbSNP or gnomAD databases and thus represents a novel private variant. Four missense variants were identified exclusively in controls and were entirely absent in CPP patients: p.Arg237Gln (Exon 4; rs146498360), p.Ile1689Phe (Exon 28; rs60854092), p.Arg1786Gln (Exon 30; rs587634422), and p.Arg2003Gln (Exon 33; rs142978073). The remaining four variants were present at comparably low frequencies in both groups: p.Ile681Asn (M3), p.Pro939Leu (M4), p.Arg1260His (M5), and p.Arg2298Trp (M11). Across all missense variants, the affected protein domains encompassed EGF-like repeats (M1–M5), the calcium-binding EGF-like repeat region (M5–M6), the heterodimerization domain (HD; M7–M8), the ANK domain (M9–M10), and the transcriptional activation domain (TAD; M11).

### 2.4. Statistically Significant Variant: p.Ile1689Phe

Among the 55 *NOTCH2* variants identified in this study, p.Ile1689Phe showed the only statistically significant frequency imbalance between groups (control allele frequency: 13/200 = 6.5%; CPP allele frequency: 0/200 = 0%; Fisher’s exact *p* = 0.0002; Bonferroni-corrected significance threshold *p* < 0.000909). This observed difference for p.Ile1689Phe remained significant after Bonferroni correction for 55 simultaneous comparisons (adjusted threshold *p* < 0.000909); no other variant met the corrected significance threshold. All 13 control carriers were heterozygous, and all had documented menarche at or after 12 years of age, confirming normal pubertal progression by questionnaire-based retrospective assessment. Individual gonadotropin data were not available for control subjects, as GnRH stimulation testing was not performed in this population; accordingly, a definitive protective interpretation at the individual level is not possible from the present data. This variant was absent from the CPP group, a distribution that may be consistent with a protective effect requiring independent replication; no causal or protective interpretation is warranted from the present data alone, and this finding should be regarded as preliminary. Three missense variants, p.Thr235Ser, p.Ala1361Thr, and p.Ala1992Val, were identified exclusively in the CPP group, each in a single allele; given this singleton status, these variants are reported descriptively as candidates for future validation, and no pathogenic interpretation is warranted from the present data.

### 2.5. Synonymous and Non-Coding Variants

A total of 13 synonymous variants were identified across the *NOTCH2* coding sequence, spanning Exon 1 through Exon 34. None demonstrated a statistically significant difference in allele frequency between CPP patients and controls (all *p* > 0.05). Representative synonymous variants, selected on the basis of allele frequency, domain location, or novelty, are listed in Table 3 (S1–S4); these include p.Arg5Arg (rs4021006; 4.0% vs. 5.0%), p.Ser1338Ser (rs17024525; 14.5% in both groups), p.Val1818Val (novel; RAM domain), and p.Leu2141Leu (rs3795666; 4.0% vs. 4.5%). In addition, 21 intronic variants distributed across introns 4 through 30 and one INDEL (poly-T repeat expansion at the Intron 6/Exon 7 boundary; chr1:119,968,316) were identified; none reached statistical significance. The complete catalogue of all 13 synonymous variants, 21 intronic variants, and 1 INDEL including chromosomal positions, dbSNP identifiers, and per-group allele frequencies is provided in Supplementary Table S2.

## 3. Discussion

In the present study, we performed a comprehensive mutational analysis of the *NOTCH2* gene in 100 Korean girls with idiopathic CPP and 100 healthy female controls with a normal age of menarche. A total of 55 sequence variants were identified, including 11 missense variants, 13 synonymous substitutions, 21 intronic variants, and 1 insertion-deletion. The principal finding of this study was that the missense variant p.Ile1689Phe (rs60854092), located within the HD-C subdomain of the NOTCH2 heterodimerization (HD) domain, was entirely absent in the CPP cohort yet present in 6.5% of the control alleles, a distribution that may be consistent with a protective role against premature HPG axis activation—a hypothesis requiring independent replication before any causal inference can be drawn. In addition, three missense variants were identified exclusively in CPP patients, including the novel variant p.Ala1992Val; these CPP-exclusive variants are reported as observational findings without causal attribution of NOTCH2 coding variants to CPP susceptibility in the Korean pediatric population.

The *NOTCH2* gene, located on chromosome 1p13–p11, encodes a 2471-amino acid type I single-pass transmembrane receptor belonging to the evolutionarily conserved Notch receptor family (NOTCH1–4) [16]. The mature receptor is organized into several functionally distinct extracellular and intracellular modules: 36 tandem epidermal growth factor (EGF)-like repeats at the N-terminus that mediate ligand binding, three Lin-12/Notch repeat (LNR) modules constituting the negative regulatory region (NRR), a bipartite HD subdivided into HD-N and HD-C, and a transmembrane segment [17]. The intracellular portion includes a RAM domain, seven ANK repeats essential for ternary complex formation with the transcriptional effectors CSL [CBF1 (C promoter-binding factor 1)/RBPJκ/Suppressor of Hairless/Lag-1] and mastermind-like (MAML), a TAD, and a C-terminal PEST (proline-, glutamic acid-, serine-, and threonine-rich) domain that targets the receptor for proteasomal degradation [16,17]. Canonical Notch signaling is initiated when a Jagged or Delta-like ligand on an adjacent cell binds to the EGF-like repeats, inducing conformational rearrangement of the NRR and sequential proteolytic cleavages: ADAM10/TACE at the S2 site and γ-secretase at the S3 site, releasing the Notch intracellular domain (NICD) [18]. The released NICD translocates to the nucleus and converts CSL from a transcriptional repressor to an activator, driving expression of downstream targets including the HES and HEY family of basic helix-loop-helix transcription factors [16]. Pathogenic *NOTCH2* variants are known to underlie both loss-of-function states (Alagille syndrome type 2, characterized by hepatic and cardiovascular malformations) and gain-of-function states: truncating mutations in exon 34 eliminate the PEST domain, preventing ubiquitin-mediated NICD degradation and producing constitutively elevated Notch2 signaling, the molecular basis of Hajdu–Cheney syndrome (HCS), a rare skeletal dysplasia with acro-osteolysis and progressive osteoporosis [18,19]. The broad tissue expression of *NOTCH2*, encompassing the hypothalamus, anterior pituitary, and GnRH neuronal system, positions this receptor as a plausible regulator of reproductive neuroendocrinology [11].

The role of Notch signaling in pubertal regulation has gained substantial attention over the past decade, particularly following the identification of mutations in the Notch pathway–related gene *DLK1* as a monogenic cause of CPP [20]. *DLK1*, a maternally imprinted gene on chromosome 14q32.2, encodes a membrane-bound and soluble non-canonical Notch ligand that suppresses canonical Notch signaling; accordingly, paternally inherited loss-of-function mutations in *DLK1* lead to disinhibition of Notch signaling and premature HPG axis activation [21]. In the pituitary, NOTCH2 is expressed in progenitor cells during organogenesis, and experimental evidence from transgenic mouse models has demonstrated that persistent NOTCH2 activation delays gonadotrope differentiation by maintaining pituitary progenitors in an undifferentiated state, thereby preventing the timely expression of the gonadotropin subunits LH and FSH [22,23]. Additionally, Notch ligand JAG1 has been shown to regulate GnRH neuron migration from the olfactory placode to the hypothalamus; loss-of-function *JAG1* variants have been identified in patients with congenital hypogonadotropic hypogonadism, indicating that the Jag1/Notch axis participates in the early ontogeny of the GnRH neuronal network [24]. Aberrant Notch signaling has thus been proposed as a unifying mechanism capable of disrupting pubertal timing at multiple levels of the HPG axis [11]. Against this background, the present finding that p.Ile1689Phe is enriched in controls in this cohort (6.5% control allele frequency) while being entirely absent from all 200 CPP alleles represents a noteworthy preliminary observation warranting further investigation.

Residue Ile1689 resides within the HD-C subdomain of the NOTCH2 receptor, the portion of the HD that remains associated with the NTMIC fragment after furin-mediated S1 cleavage and forms the structural core of the autoinhibitory NRR [17]. The substitution of isoleucine with phenylalanine at position 1689 within the NOTCH2 HD-C subdomain may influence the conformational stability of the NRR by introducing a bulkier aromatic side chain into a hydrophobic core; this structural hypothesis requires confirmation by thermal stability assays, structural modeling, and Notch signaling reporter assays before any functional conclusion can be drawn. The present data do not establish a causal or protective role of p.Ile1689Phe against premature HPG axis activation; rather, the observed frequency imbalance is a preliminary signal that requires independent replication in larger, multi-ethnic cohorts before any causal inference can be drawn. The relatively high population frequency of this variant in East Asian individuals (gnomAD East Asian frequency of approximately 5 to 7 percent), combined with its Variant of Uncertain Significance (VUS) designation in ClinVar, is more consistent with a common-variant contribution to a quantitative trait such as pubertal timing than with a Mendelian protective allele.

In addition to the principal finding, three missense variants such as p.Thr235Ser, p.Ala1361Thr, and p.Ala1992Val were identified exclusively in the CPP cohort, each observed in a single allele; given this singleton status, none of these variants can be distinguished from stochastic sampling variation at the present sample size, and no pathogenic interpretation is warranted from the current data alone. The most structurally notable of these is the novel variant p.Ala1992Val: residue Ala1992 resides within the intracellular ANK domain of NOTCH2, a module comprising seven stacked ANK repeats that constitutes the primary protein–protein interaction interface for assembly of the transcriptionally active CSL–NICD–MAML ternary complex [16,17]; this domain localization is reported as a point of biological interest, but the singleton status of this variant precludes any mechanistic inference, and in vitro Notch transcriptional reporter assays and NICD–MAML interaction studies will be required before any functional conclusion can be drawn [17]. All three CPP-exclusive variants are reported for completeness and as candidates for future functional validation and replication in independent cohorts. Collectively, the observation of multiple rare *NOTCH2* coding variants restricted to the CPP group raises the hypothesis that *NOTCH2* may contribute to CPP susceptibility within an oligogenic framework—a concept increasingly supported by whole-exome sequencing studies demonstrating that rare variants across multiple CPP-related loci can co-occur in individual patients and collectively modulate the threshold for pubertal onset [23,24,25,26]; testing this hypothesis will require larger cohorts incorporating family-based segregation analysis, functional validation, and multi-ethnic replication.

ClinVar classifications were retrieved for all 11 missense variants identified in this study and are now provided in full in Supplementary Table S2. Of these, five were classified as Benign or Likely Benign, five as Variants of Uncertain Significance (VUS), and one (p.Ala1992Val) was absent from ClinVar at the time of analysis (April 2026). The predominance of benign and uncertain classifications is consistent with the recognized difficulty of interpreting NOTCH2 missense variants under Mendelian disease frameworks [27,28]. The principal finding of this study, p.Ile1689Phe (rs60854092), carries a VUS designation in ClinVar in the context of Alagille syndrome, with no prior entry related to CPP or pubertal timing. Under ACMG/AMP criteria, a VUS reflects insufficient evidence for reclassification in the Mendelian context and does not preclude a contribution to quantitative trait variation [28]; the gnomAD v3.1.2 East Asian allele frequency of approximately 5–7% is incompatible with full-penetrance Mendelian disease but is consistent with common-variant effects on a polygenic trait such as pubertal timing. The novel variant p.Ala1992Val was classified as VUS by InterVar based on PM2 and PP3 criteria; given its singleton observation in a single CPP allele, it is reported as a candidate for future functional characterization without pathogenic interpretation. For the remaining Benign and Likely Benign variants, their population frequencies and absence of statistically significant group differences in the present study are consistent with these ClinVar designations.

The novel synonymous variant p.Val1818Val (c.5454G>C; Exon 30) was identified in a single heterozygous control individual and was absent from all CPP alleles examined. In silico splicing analysis was performed to evaluate potential effects on pre-mRNA splicing. Human Splicing Finder (HSF v3.1) [29] predicted a modest reduction in the composite ESE score at the variant position, consistent with partial disruption of a predicted SRSF1-binding motif, without predicted disruption of the canonical donor splice site. SpliceAI [30] yielded sub-threshold scores (donor gain: 0.08; donor loss: 0.06; significance threshold: 0.2). The variant is located approximately 8 bp from the Exon 30/Intron 30 boundary, outside the canonical MaxEntScan 9-mer scoring window [29], and the MaxEntScan delta score (−0.3) did not reach the 5′ donor disruption threshold (−3.0). The available in silico evidence does not support a significant effect on canonical NOTCH2 pre-mRNA splicing. Definitive characterization of any residual splicing consequence would require a minigene functional assay [31], which is beyond the scope of the present study.

Several limitations of this study should be acknowledged. First, the cohort was restricted to Korean girls, which limits the generalizability of the allele frequency data—particularly for p.Ile1689Phe—to other ethnic populations; independent, multi-ethnic replication studies are required before broader conclusions can be drawn [2]. Second, although the sample size was sufficient to detect the statistically robust p.Ile1689Phe association, it remains underpowered for variants with small-to-moderate effect sizes, and the absence of family-based segregation analysis precluded determination of de novo versus inherited origin for the CPP-exclusive variants, a step that is increasingly considered essential for establishing causality in genetic studies of CPP [26,32]. Third, functional validation of the identified missense variants was not performed; the biological significance of all variants including the novel p.Ala1992Val currently rests on statistical and in silico evidence, and cellular or animal model experiments are warranted to confirm the proposed mechanistic hypotheses [11,23]. Fourth, population stratification cannot be entirely excluded. Although all participants were recruited from the same institution and self-identified as Korean, subtle genetic substructure between the clinical cohort and the separately recruited healthy controls cannot be entirely ruled out without genome-wide ancestry-informative markers or principal component analysis [33]. The observed control allele frequency of p.Ile1689Phe (6.5%) is broadly consistent with gnomAD v3.1.2 East Asian population data (~5–7%) [34], providing contextual reassurance that the control frequency is not anomalous; nonetheless, formal stratification correction using genome-wide SNP arrays should be incorporated in future replication studies. Fifth, the analysis was confined to coding regions and selected intronic sequences; epigenetic modifications, copy number variations, and potential splicing effects of deep intronic variants in the *NOTCH2* locus were not evaluated, leaving open the possibility that additional non-coding regulatory mechanisms contribute to *NOTCH2* dysregulation in CPP [26]. Nevertheless, this study represents the first systematic candidate-gene analysis of *NOTCH2* in a well-characterized cohort of Korean girls with idiopathic CPP, employing a matched case–control design with comprehensive sequencing of the full coding region. The identification of a statistically significant protective variant and multiple CPP-exclusive missense variants provides a meaningful foundation for future functional and multi-ethnic investigations of the Notch signaling pathway in human pubertal timing.

In conclusion, this study provides the first systematic candidate-gene analysis of *NOTCH2* in Korean girls with idiopathic CPP and identifies a statistically significant frequency imbalance for the HD-C subdomain variant p.Ile1689Phe, which was absent from the CPP cohort while present in 6.5% of control alleles; this distribution raises the hypothesis that certain *NOTCH2* HD-C subdomain variants may modulate the threshold for HPG axis activation, a possibility that requires independent replication and functional validation before any causal inference can be drawn. The novel ANK domain variant p.Ala1992Val and two additional CPP-exclusive missense variants were also observed; given their singleton status, no pathogenic interpretation is made, but they are reported as candidates for future validation in the context of *NOTCH2* biology. Together, these preliminary findings expand the candidate genetic landscape of idiopathic CPP beyond the established monogenic causes—*MKRN3*, *DLK1*, *KISS1*, and *KISS1R*—and raise the hypothesis that *NOTCH2* coding variants may contribute to the regulation of pubertal timing within the Notch signaling pathway. All findings are hypothesis-generating; future studies incorporating functional validation, family-based designs, genome-wide approaches, and multi-ethnic replication cohorts will be essential to fully delineate the contribution of *NOTCH2* to the complex genetic architecture of human pubertal timing.

## 4. Materials and Methods

### 4.1. Subjects and Study Design

This single-center, prospective case–control study enrolled 100 girls diagnosed with idiopathic CPP at Ajou University Hospital between March 2016 and December 2018. The diagnosis of CPP was established according to the following criteria: (1) objective breast development (Tanner stage ≥ II) before the age of 8 years; (2) a bone age advance of more than 1 year relative to chronological age, as assessed by the method of Greulich and Pyle [35]; and (3) a pubertal luteinizing hormone (LH) response to the GnRH stimulation test, defined as a peak LH level exceeding 5.0 IU/L on immunoradiometric assay (IRMA). Prior to enrollment, all 100 CPP patients underwent targeted Sanger sequencing of the established monogenic CPP genes *MKRN3*, *DLK1*, *KISS1*, and *KISS1R*. No pathogenic or likely pathogenic variants (as defined by ACMG/AMP 2015 criteria) were identified in any patient for these genes. Patients were subsequently enrolled in the *NOTCH2* candidate-gene study on the basis of this exclusion. Of the 100 enrolled patients, 13 (13%) had a documented family history of CPP. Subjects with a history of prior hormonal treatment or known chromosomal abnormalities were excluded, as were cases of precocious puberty attributable to an identifiable organic etiology, including intracranial tumor and prior cranial irradiation. Thyroid function was assessed in all subjects by measurement of serum T4 and TSH levels to exclude hypothyroidism as a contributing factor.

Control subjects were healthy female volunteers recruited from Ajou University Hospital, Suwon, Republic of Korea, during the same enrollment period (March 2016–December 2018). Inclusion criteria were as follows: (i) females with age ≥ 12 years at enrollment; (ii) documented menarche at age ≥ 12.0 years, ascertained by structured questionnaire administered at enrollment; (iii) height and weight within ±2 SDS of age- and sex-specific reference values according to the 2017 Korean National Growth Charts [15]; and (iv) Tanner breast stage IV–V confirmed by physical examination at enrollment, consistent with active or recently completed pubertal maturation. Girls with any known chronic illness, endocrine disorder, or prior pubertal evaluation were excluded. Because all enrolled controls demonstrated age-appropriate growth and normal pubertal progression, GnRH stimulation testing was not clinically indicated and was therefore not performed; bone age radiography was performed in all control subjects and confirmed skeletal maturation consistent with chronological age. Longitudinal follow-up was not available in this retrospective cohort; accordingly, the possibility that a small proportion of controls may have experienced early puberty cannot be entirely excluded; however, unrecognized true CPP in this group is considered unlikely, given that all participants met the strict inclusion criterion of menarche at age > 12.0 years and demonstrated age-appropriate growth and Tanner breast stage IV–V at enrollment. The study protocol was approved by the Institutional Review Board (IRB) of Ajou University Hospital (Ethics Committee Name: IRB of Ajou University Hospital, Approval Code: AJOUIRB-GEN-2016-210, Approval Date: 8 September 2016), and written informed consent was obtained from all participants or their legal guardians prior to enrollment. All genomic DNA specimens and accompanying clinical data used in this study were obtained from the Ajou University Medical Center Human Genome Resource Bank, which provided DNA specimens from 100 patients with idiopathic CPP and 100 healthy control subjects.

Height and weight were measured using a Harpenden stadiometer (Holtain Ltd., Crymych, Wales, UK) and a calibrated scale, respectively. Sexual maturation was staged according to the Tanner criteria (stages II–V) [36]. Body mass index (BMI) was calculated as weight (kg) divided by height squared (m^2^). SDS for height, weight, and BMI were derived using the Lambda–Mu–Sigma (LMS) method in accordance with the 2017 Korean National Growth Charts [15]. Bone age advancement (BA–CA) was calculated as the difference between radiological bone age and chronological age. GnRH stimulation tests were performed between 12:30 and 16:00. Serum LH and follicle-stimulating hormone (FSH) levels were measured at 0, 30, 45, 60, and 90 min following an intravenous bolus injection of 100 µg synthetic GnRH (Relefact; Sanofi-Aventis, Frankfurt, Germany). Both LH and FSH were quantified by IRMA using commercial kits (BioSource, Nivelles, Belgium), with detection limits of 0.1 IU/L for LH and 0.2 IU/L for FSH. Predicted adult height (PAH) was estimated by the Bayley–Pinneau method at the time of diagnosis [37].

### 4.2. Genomic DNA Extraction

Genomic DNA specimens for all CPP patients and control subjects were obtained from the Ajou University Medical Center Human Genome Resource Bank. DNA had been extracted from peripheral blood leukocytes by the Biobank using a commercially available DNA isolation kit (QIAamp DNA Blood Mini Kit; QIAGEN GmbH, Hilden, Germany) in accordance with the manufacturer’s instructions. Upon receipt, DNA concentration and purity were verified by UV spectrophotometry (NanoDrop; Thermo Fisher Scientific, Wilmington, DE, USA), and DNA specimens were stored at −20 °C until use.

### 4.3. NOTCH2 Sequencing Strategy

The *NOTCH2* gene (RefSeq transcript accession NM_024408.6; chromosomal location 1p13–p11) encodes a type I transmembrane receptor comprising multiple functional domains, including extracellular epidermal growth factor (EGF)-like repeats, calcium-binding EGF-like (cbEGF) repeats, the heterodimerization (HD) domain subdivided into HD-N and HD-C subdomains, the NRR, the transmembrane domain, the RBP-J-associated molecule (RAM) domain, the ANK domain, and the C-terminal TAD [5]. To achieve comprehensive coverage of all potential coding variants, all 34 coding exons of *NOTCH2* and their flanking intronic sequences (minimum 20 bp on each side of each exon–intron boundary) were amplified and subjected to bidirectional Sanger sequencing. A total of 37 overlapping amplicons were designed to ensure complete coverage: exons 1 through 33 were each covered by a single amplicon (amplicons *NOTCH2-1* through *NOTCH2-33*), while the large exon 34 was covered by four consecutive overlapping amplicons (*NOTCH2-34-1* through *NOTCH2-34-4*). The complete list of primer sequences, amplicon sizes, and annealing temperatures is provided in Supplementary Table S1.

### 4.4. PCR Amplification

All polymerase chain reaction (PCR) amplifications were performed using a premixed polymerase system (Premix Taq; TaKaRa Bio Inc., Kusatsu, Japan). Each 20 µL reaction contained 10 µL of Premix Taq, 1 µL of each forward and reverse primer (10 pmol/µL), 1 µL of template genomic DNA (~50 ng), and 7 µL of nuclease-free water. The standard thermocycling program consisted of an initial denaturation at 95 °C for 5 min, followed by 35 cycles of denaturation at 95 °C for 30 s, primer annealing at the amplicon-specific temperature for 30 s, and extension at 72 °C for 1 min, with a final extension at 72 °C for 5 min. Annealing temperatures were optimized for each amplicon and ranged from 58 °C to 62 °C (median 60 °C), as specified in Supplementary Table S1. After amplification, PCR products were resolved by electrophoresis on 1.5% agarose gels stained with ethidium bromide to confirm product size and amplification fidelity prior to sequencing. Selected amplicons for which confirmatory sequencing of identified variants was required were re-amplified using an alternative internal reverse primer (e.g., NOTCH2-11R1: 5′-TGA CTT CTC CAC TGG CTA GG-3′) to provide independent sequence confirmation.

### 4.5. Sanger Sequencing and Variant Detection

Purified PCR products were subjected to cycle sequencing using the BigDye Terminator v3.1 Cycle Sequencing Kit (Applied Biosystems, Foster City, CA, USA) according to the manufacturer’s protocol, with the same primers used for amplification to enable bidirectional sequencing of all amplicons. Sequencing products were resolved by capillary electrophoresis on an ABI 3130xl Genetic Analyzer (Applied Biosystems), and electropherograms were analyzed using Sequencing Analysis software v5.2. All sequence traces were aligned to the *NOTCH2* reference sequence (NM_024408.6; genomic reference NC_000001.11) using Sequencher v5.4 (Gene Codes Corporation, Ann Arbor, MI, USA). Sequence variants were initially called by automated alignment and subsequently confirmed by visual inspection of bidirectional electropherograms by two independent reviewers. All identified variants were verified by re-sequencing from an independent PCR amplification of the relevant amplicon. Samples carrying variants of interest were sequenced in both forward and reverse directions, and all positive findings were repeated from a freshly prepared aliquot of genomic DNA to exclude PCR-introduced artefacts.

### 4.6. Variant Annotation and Classification

Nucleotide positions are reported according to the NM_024408.6 coding sequence, with nucleotide numbering beginning at the adenine of the ATG translation initiation codon (c.1). Amino acid positions are numbered according to the canonical NOTCH2 protein (UniProt accession Q04721). Identified sequence variants were cross-referenced against the Single Nucleotide Polymorphism database (dbSNP, build 156; National Center for Biotechnology Information), the Genome Aggregation Database (gnomAD v3.1.2) [34], the ClinVar database, and the Human Gene Mutation Database (HGMD) to assess prior documentation, allele frequency in population cohorts, and previously reported clinical significance. Variants absent from all reference databases were designated as novel (private) variants. Chromosomal coordinates were mapped to the GRCh38/hg38 reference assembly. All missense variants were classified according to the American College of Medical Genetics and Genomics and the Association for Molecular Pathology (ACMG/AMP) variant interpretation guidelines using the ClinVar Variant Interpreter and InterVar software [38]. The functional domain location of each variant was assigned based on the established structural annotation of *NOTCH2*, including the extracellular EGF-like repeat region (residues 1–1390), the HD-N (residues ~1447–1579) and HD-C (residues ~1580–1700; boundary revised from the previous submission per UniProt Q04721 domain annotation) subdomains, the transmembrane domain, the RAM domain, the ANK domain (seven ankyrin repeats, residues ~1851–2126), and the C-terminal TAD [39].

### 4.7. In Silico Pathogenicity Prediction

The functional impact of all identified missense variants was evaluated using multiple complementary in silico prediction tools: SIFT (Sorting Intolerant From Tolerant; https://siftdna.org/, accessed on 17 July 2026) [40], PolyPhen-2 (Polymorphism Phenotyping v2; https://genetics.bwh.harvard.edu/pph2/, accessed on 17 July 2026) [41], MutationTaster (https://www.mutationtaster.org/, accessed on 17 July 2026) [42], and the Combined Annotation–Dependent Depletion score (CADD v1.6; https://cadd.gs.washington.edu/, accessed on 17 July 2026) [43]. Variants yielding CADD scaled scores ≥ 20 were considered potentially deleterious. Conservation of affected residues across vertebrate species was assessed using the UCSC Genome Browser 100-vertebrate PhyloP and PhastCons scores. For synonymous variants and intronic variants within the canonical splice site (±2 bp), potential effects on pre-mRNA splicing were assessed using the Human Splicing Finder (HSF v3.1; https://umd.be/hsf/, accessed on 17 July 2026) [29] and MaxEntScan [44]. Structural modeling of the HD domain variant p.Ile1689Phe was performed using the AlphaFold2-predicted structure of *NOTCH2* (AF-Q04721-F1) [45] to evaluate potential effects on NRR conformation and autoinhibitory packing.

### 4.8. Statistical Analysis

Hardy–Weinberg equilibrium (HWE) was assessed separately in the CPP group and the control group for each variant with a minor allele count of ≥2 in that group, using the chi-square goodness-of-fit test; singleton variants (allele count = 1) were excluded from HWE testing, and deviation from HWE was defined as *p* < 0.05. Allele and genotype frequencies for each identified variant were compared between CPP patients and controls using the chi-square test or, where one or more cells had an expected count of fewer than five, Fisher’s exact test. For multiple comparisons, Bonferroni correction was applied; the adjusted significance threshold for 55 simultaneous comparisons was *p* < 0.000909 (0.05/55). Both uncorrected and Bonferroni-corrected *p* values are reported. When a statistically significant difference in allele frequency was identified after Bonferroni correction, independent-samples *t*-tests were applied to compare relevant clinical and hormonal variables (e.g., peak LH, bone age advancement) between subjects harboring the variant and those without it. Results are expressed as means ± standard deviation (SD) unless otherwise stated. All statistical analyses were performed using R software version 4.5.1 (R Foundation for Statistical Computing, Vienna, Austria). A two-tailed *p* < 0.05 was considered statistically significant for secondary analyses; for primary allele frequency comparisons, the Bonferroni corrected threshold of *p* < 0.000909 was applied.

## Figures and Tables

**Table 1:** Clinical and laboratory characteristics of girls with central precocious puberty.

Characteristics	CPP Patients (n = 100)
Age at diagnosis (years)	6.75 ± 0.47
Height (cm)	123.43 ± 4.92
Height SDS	0.91 ± 0.93
Weight (kg)	26.12 ± 4.50
Weight SDS	0.79 ± 1.00
BMI (kg/m^2^)	17.06 ± 2.18
BMI SDS	0.45 ± 1.14
Father’s height (cm)	173.02 ± 5.04
Mother’s height (cm)	159.94 ± 4.98
Target height (cm)	159.98 ± 3.61
Target height SDS	−0.16 ± 0.72
Tanner stage (breast), n (%)	
II	88 (88%)
III	12 (12%)
Bone age (years)	8.83 ± 0.94
BA–CA (years)	2.05 ± 0.81
PAH (cm)	150.43 ± 6.43
PAH SDS	−2.18 ± 1.41
Basal LH (IU/L)	0.20 ± 0.27
Peak LH (IU/L)	8.05 ± 4.45
Basal FSH (IU/L)	2.11 ± 0.92
Peak FSH (IU/L)	16.70 ± 5.44
Basal estradiol, E2 (pg/mL)	25.41 ± 24.56

Values are expressed as mean ± standard deviation (SD) unless otherwise stated. SDS, standard deviation score; BA, bone age; CA, chronological age; BA–CA, bone age advancement; PAH, predicted adult height; LH, luteinizing hormone; FSH, follicle-stimulating hormone; E2, estradiol. SDS values were calculated based on the Korean National Growth Charts (2017) [15].

**Table 2:** Clinical and laboratory characteristics of controls.

Characteristics	Controls (n = 100)
Age at enrollment (years)	13.91 ± 1.03
Age at menarche (years)	12.51 ± 0.63
Height (cm)	157.52 ± 5.38
Height SDS	0.04 ± 0.95
Weight (kg)	47.69 ± 6.95
Weight SDS	−0.33 ± 0.88
BMI (kg/m^2^)	19.20 ± 2.50
BMI SDS	−0.44 ± 0.95
Father’s height (cm)	172.85 ± 5.40
Mother’s height (cm)	160.49 ± 4.40
Target height (cm)	160.17 ± 3.65
Target height SDS	−0.17 ± 0.68
Tanner stage (breast), n (%)	
IV	41 (41%)
V	59 (59%)
Tanner stage (pubic hair), n (%)	
I	4 (4%)
II	36 (36%)
III	36 (36%)
IV	18 (18%)
V	6 (6%)
Bone age (years)	14.69 ± 1.44

Continuous variables are expressed as Mean ± SD and categorical variables are expressed as number (%). Target height and Target height standard deviation score (SDS) were determined using mid-parental height as 0.5 × (paternal height + maternal height − 13) cm. SDS, standard deviation score. SDS values were calculated based on the Korean National Growth Charts (2017) [15].

**Table 3:** Allele frequencies of NOTCH2 missense and representative synonymous variants in girls with central precocious puberty and controls.

No.	Exon	Genetic Variant	Amino Acid Change	Protein Domain	ClinVar	dbSNP	Controls (n = 100) n/200 Alleles (%)	CPP (n = 100) n/200 Alleles (%)	Uncorrected *p*	Corrected *p*
Selected synonymous variants										
S1	Ex1	c.15C>T	p.Arg5Arg	Extracellular (N-terminal)	B	rs4021006	8/200 (4.0%)	10/200 (5.0%)	0.660	1.000
S2	Ex1	c.45C>T	p.Leu15Leu	EGF-like repeat domain	LB	rs782485593	0/200 (0.0%)	1/200 (0.5%)	1.000	1.000
S3	Ex10	c.1653G>A	p.Pro551Pro	cbEGF region	LB	rs187731832	0/200 (0.0%)	1/200 (0.5%)	1.000	1.000
S4	Ex19	c.3034T>C	p.Leu1012Leu	cbEGF-like repeat domain	B	rs77194332	5/200 (2.5%)	5/200 (2.5%)	1.000	1.000
S5	Ex25	c.4014C>T	p.Ser1338Ser	cbEGF-like repeat domain	B	rs17024525	29/200 (14.5%)	29/200 (14.5%)	1.000	1.000
S6	Ex25	c.4305G>A	p.Arg1435Arg	NRR	LB	rs6692009	0/200 (0.0%)	1/200 (0.5%)	1.000	1.000
S7	Ex30	c.5454G>C	p.Val1818Val	RAM domain	NE	Novel	1/200 (0.5%)	0/200 (0.0%)	1.000	1.000
S8	Ex34	c.6045T>C	p.Phe2015Phe	ANK domain	LB	rs766885963	0/200 (0.0%)	1/200 (0.5%)	1.000	1.000
S9	Ex34	c.6150T>A	p.Ala2050Ala	ANK domain	LB	rs764208739	0/200 (0.0%)	1/200 (0.5%)	1.000	1.000
S10	Ex34	c.6222C>T	p.Thr2074Thr	ANK domain	LB	rs764335447	1/200 (0.5%)	0/200 (0.0%)	1.000	1.000
S11	Ex34	c.6421C>T	p.Leu2141Leu	TAD	B	rs3795666	8/200 (4.0%)	9/200 (4.5%)	1.000	1.000
S12	Ex34	c.7263T>A	p.Ser2421Ser	TAD	LB	rs369912969	0/200 (0.0%)	1/200 (0.5%)	1.000	1.000
S13	Ex34	c.7341T>A	p.Gly2447Gly	TAD	B	rs6685892	6/200 (3.0%)	3/200 (1.5%)	0.510	1.000
Missense variants										
M1	Ex4	c.703A>T	p.Thr235Ser †	EGF-like repeat (N-terminal)	LB	rs200464440	0/200 (0%)	1/200 (0.5%)	1.000	1.000
M2	Ex4	c.710G>A	p.Arg237Gln ‡	EGF-like repeat	VUS	rs146498360	2/200 (1.0%)	0/200 (0%)	0.498	1.000
M3	Ex13	c.2042T>A	p.Ile681Asn	EGF-like/LNR boundary	LB	rs74882029	1/200 (0.5%)	1/200 (0.5%)	1.000	1.000
M4	Ex18	c.2816C>T	p.Pro939Leu	LNR module A	VUS	rs201100122	1/200 (0.5%)	1/200 (0.5%)	1.000	1.000
M5	Ex23	c.3779G>A	p.Arg1260His	EGF-like/NRR proximal	LB	rs75423398	12/200 (6.0%)	6/200 (3.0%)	0.180	1.000
M6	Ex25	c.4081G>A	p.Ala1361Thr †	cbEGF-like repeat 35	VUS	rs138567855	0/200 (0%)	1/200 (0.5%)	1.000	1.000
M7	Ex28	c.5065A>T	p.Ile1689Phe ‡*	HD-C subdomain (NRR)	VUS	rs60854092	13/200 (6.5%)	0/200 (0%)	0.0002 *	0.011 *
M8	Ex30	c.5357G>A	p.Arg1786Gln ‡	Transmembrane proximal	VUS	rs587634422	1/200 (0.5%)	0/200 (0%)	1.000	1.000
M9	Ex33	c.5975C>T	p.Ala1992Val †§	ANK domain	NE	Novel §	0/200 (0%)	1/200 (0.5%)	1.000	1.000
M10	Ex33	c.6008G>A	p.Arg2003Gln ‡	ANK domain	LB	rs142978073	1/200 (0.5%)	0/200 (0%)	1.000	1.000
M11	Ex34	c.6892C>T	p.Arg2298Trp	PEST domain	VUS	rs1270274564	1/200 (0.5%)	1/200 (0.5%)	1.000	1.000

All allele frequencies were calculated based on 200 total alleles per group (100 diploid individuals; all variants were heterozygous). *p* values were determined by two-sided Fisher’s exact test. † Variant detected exclusively in CPP patients (absent in all controls). ‡ Variant detected exclusively in controls (absent in all CPP patients). * *p* < 0.05; statistically significant. § Novel variant not registered in dbSNP or gnomAD databases (as of April 2026). dbSNP: Single Nucleotide Polymorphism database; CPP, central precocious puberty; EGF, epidermal growth factor; cbEGF, calcium-binding EGF-like repeat; NRR, negative regulatory region; LNR, Lin-12/Notch repeat domain; ANK, ankyrin repeat domain; TAD, transcriptional activation domain; HD-C, heterodimerization domain C-terminal subdomain.

## Data Availability

The data presented in this study are available from the corresponding author upon reasonable request. The data are not publicly available due to ethical and privacy considerations.

## Supplementary Materials

The following supporting information can be downloaded at: https://www.mdpi.com/article/10.3390/ijms27156686/s1.

## References

[1] Cheuiche A.V., da Silveira L.G., de Paula L.C.P., Lucena I.R.S., Silveiro S.P. (2021). Diagnosis and management of precocious sexual maturation: An updated review. Eur. J. Pediatr..

[2] Shim Y.S., Lee H.S., Hwang J.S. (2022). Genetic factors in precocious puberty. Clin. Exp. Pediatr..

[3] Jeong H.R., Hwang I.T. (2024). The role of MicroRNAs as fine-tuners in the onset of puberty: A comprehensive review. Ann. Pediatr. Endocrinol. Metab..

[4] Cantas-Orsdemir S., Eugster E.A. (2019). Update on central precocious puberty: From etiologies to outcomes. Expert Rev. Endocrinol. Metab..

[5] Li C., Lu W., Yang L., Li Z., Zhou X., Guo R., Wang J., Wu Z., Dong Z., Ning G. (2020). MKRN3 regulates the epigenetic switch of mammalian puberty via ubiquitination of MBD3. Natl. Sci. Rev..

[6] Livadas S., Chrousos G.P. (2019). Molecular and Environmental Mechanisms Regulating Puberty Initiation: An Integrated Approach. Front. Endocrinol..

[7] Roberts S.A., Kaiser U.B. (2020). GENETICS IN ENDOCRINOLOGY: Genetic etiologies of central precocious puberty and the role of imprinted genes. Eur. J. Endocrinol..

[8] Canton A.P.M., Krepischi A.C.V., Montenegro L.R., Costa S., Rosenberg C., Steunou V., Sobrier M.L., Santana L., Honjo R.S., Kim C.A. (2021). Insights from the genetic characterization of central precocious puberty associated with multiple anomalies. Hum. Reprod..

[9] Neocleous V., Fanis P., Toumba M., Gorka B., Kousiappa I., Tanteles G.A., Iasonides M., Nicolaides N.C., Christou Y.P., Michailidou K. (2021). Pathogenic and Low-Frequency Variants in Children With Central Precocious Puberty. Front. Endocrinol..

[10] Macedo D.B., Kaiser U.B. (2019). DLK1, Notch Signaling and the Timing of Puberty. Semin. Reprod. Med..

[11] Shim Y.S., Lee H.S., Hwang J.S. (2022). Aberrant Notch Signaling Pathway as a Potential Mechanism of Central Precocious Puberty. Int. J. Mol. Sci..

[12] Montenegro L., Labarta J.I., Piovesan M., Canton A.P.M., Corripio R., Soriano-Guillén L., Travieso-Suárez L., Martín-Rivada Á., Barrios V., Seraphim C.E. (2020). Novel Genetic and Biochemical Findings of DLK1 in Children with Central Precocious Puberty: A Brazilian-Spanish Study. J. Clin. Endocrinol. Metab..

[13] Lee H.S., Jeong H.R., Rho J.G., Kum C.D., Kim K.H., Kim D.W., Cheong J.Y., Jeong S.Y., Hwang J.S. (2020). Identification of rare missense mutations in NOTCH2 and HERC2 associated with familial central precocious puberty via whole-exome sequencing. Gynecol. Endocrinol..

[14] Dauber A., Cunha-Silva M., Macedo D.B., Brito V.N., Abreu A.P., Roberts S.A., Montenegro L.R., Andrew M., Kirby A., Weirauch M.T. (2017). Paternally Inherited DLK1 Deletion Associated With Familial Central Precocious Puberty. J. Clin. Endocrinol. Metab..

[15] Kim J.H., Yun S., Hwang S.S., Shim J.O., Chae H.W., Lee Y.J., Lee J.H., Kim S.C., Lim D., Yang S.W. (2018). The 2017 Korean National Growth Charts for children and adolescents: Development, improvement, and prospects. Korean J. Pediatr..

[16] Mašek J., Andersson E.R. (2017). The developmental biology of genetic Notch disorders. Development.

[17] Yamamoto S. (2020). Making sense out of missense mutations: Mechanistic dissection of Notch receptors through structure-function studies in Drosophila. Dev. Growth Differ..

[18] Simpson M.A., Irving M.D., Asilmaz E., Gray M.J., Dafou D., Elmslie F.V., Mansour S., Holder S.E., Brain C.E., Burton B.K. (2011). Mutations in NOTCH2 cause Hajdu-Cheney syndrome, a disorder of severe and progressive bone loss. Nat. Genet..

[19] Aida N., Ohno T., Azuma T. (2022). Progress and Current Status in Hajdu-Cheney Syndrome with Focus on Novel Genetic Research. Int. J. Mol. Sci..

[20] Argente J., Dunkel L., Kaiser U.B., Latronico A.C., Lomniczi A., Soriano-Guillén L., Tena-Sempere M. (2023). Molecular basis of normal and pathological puberty: From basic mechanisms to clinical implications. Lancet Diabetes Endocrinol..

[21] Lin Y., He Y., Sun W., Wang Y., Yu J. (2022). Recent advances on the relationship between the delta-like noncanonical Notch ligand 1 system and central precocious puberty. Biol. Reprod..

[22] Nantie L.B., Himes A.D., Getz D.R., Raetzman L.T. (2014). Notch signaling in postnatal pituitary expansion: Proliferation, progenitors, and cell specification. Mol. Endocrinol..

[23] Raetzman L.T., Wheeler B.S., Ross S.A., Thomas P.Q., Camper S.A. (2006). Persistent Expression of Notch2 Delays Gonadotrope Differentiation. Mol. Endocrinol..

[24] Cotellessa L., Marelli F., Duminuco P., Adamo M., Papadakis G.E., Bartoloni L., Sato N., Lang-Muritano M., Troendle A., Dhillo W.S. (2023). Defective jagged-1 signaling affects GnRH development and contributes to congenital hypogonadotropic hypogonadism. JCI Insight.

[25] Brito V.N., Canton A.P.M., Seraphim C.E., Abreu A.P., Macedo D.B., Mendonca B.B., Kaiser U.B., Argente J., Latronico A.C. (2023). The Congenital and Acquired Mechanisms Implicated in the Etiology of Central Precocious Puberty. Endocr. Rev..

[26] Canton A.P.M., Macedo D.B., Abreu A.P., Latronico A.C. (2025). Genetics and Epigenetics of Human Pubertal Timing: The Contribution of Genes Associated With Central Precocious Puberty. J. Endocr. Soc..

[27] Vandriel S.M., Li L.T., She H., Wang J.S., Loomes K.M., Piccoli D.A., Jankowska I., Czubkowski P., Gliwicz-Miedzińska D., D’Antiga L. (2025). Phenotypic Divergence of JAG1- and NOTCH2-Associated Alagille Syndrome & Disease-Specific NOTCH2 Variant Classification Guidelines. Liver Int..

[28] Kim Y.E., Ki C.S., Jang M.A. (2019). Challenges and Considerations in Sequence Variant Interpretation for Mendelian Disorders. Ann. Lab. Med..

[29] Desmet F.O., Hamroun D., Lalande M., Collod-Béroud G., Claustres M., Béroud C. (2009). Human Splicing Finder: An online bioinformatics tool to predict splicing signals. Nucleic Acids Res..

[30] Jaganathan K., Kyriazopoulou Panagiotopoulou S., McRae J.F., Darbandi S.F., Knowles D., Li Y.I., Kosmicki J.A., Arbelaez J., Cui W., Schwartz G.B. (2019). Predicting Splicing from Primary Sequence with Deep Learning. Cell.

[31] Gaildrat P., Killian A., Martins A., Tournier I., Frébourg T., Tosi M. (2010). Use of splicing reporter minigene assay to evaluate the effect on splicing of unclassified genetic variants. Methods Mol. Biol..

[32] Tinano F.R., Canton A.P.M., Montenegro L.R., de Castro Leal A., Faria A.G., Seraphim C.E., Brauner R., Jorge A.A., Mendonca B.B., Argente J. (2023). Clinical and Genetic Characterization of Familial Central Precocious Puberty. J. Clin. Endocrinol. Metab..

[33] Price A.L., Patterson N.J., Plenge R.M., Weinblatt M.E., Shadick N.A., Reich D. (2006). Principal components analysis corrects for stratification in genome-wide association studies. Nat. Genet..

[34] Karczewski K.J., Francioli L.C., Tiao G., Cummings B.B., Alfoldi J., Wang Q., Collins R.L., Laricchia K.M., Ganna A., Birnbaum D.P. (2020). The mutational constraint spectrum quantified from variation in 141,456 humans. Nature.

[35] Greulich W.W., Pyle S.I. (1959). Radiologic Atlas of Skeletal Development of the Hand and Wrist.

[36] Wheeler M.D. (1991). Physical changes of puberty. Endocrinol. Metab. Clin. N. Am..

[37] Bayley N., Pinneau S.R. (1952). Tables for predicting adult height from skeletal age: Revised for use with the Greulich-Pyle hand standards. J. Pediatr..

[38] Richards S., Aziz N., Bale S., Bick D., Das S., Gastier-Foster J., Grody W.W., Hegde M., Lyon E., Spector E. (2015). Standards and guidelines for the interpretation of sequence variants: A joint consensus recommendation of the American College of Medical Genetics and Genomics and the Association for Molecular Pathology. Genet. Med..

[39] Gordon W.R., Vardar-Ulu D., L’Heureux S., Ashworth T., Malecki M.J., Sanchez-Irizarry C., McArthur D.G., Histen G., Mitchell J.L., Aster J.C. (2009). Effects of S1 cleavage on the structure, surface export, and signaling activity of human Notch1 and Notch2. PLoS ONE.

[40] Ng P.C., Henikoff S. (2003). SIFT: Predicting amino acid changes that affect protein function. Nucleic Acids Res..

[41] Adzhubei I.A., Schmidt S., Peshkin L., Ramensky V.E., Gerasimova A., Bork P., Kondrashov A.S., Sunyaev S.R. (2010). A method and server for predicting damaging missense mutations. Nat. Methods.

[42] Schwarz J.M., Cooper D.N., Schuelke M., Seelow D. (2014). MutationTaster2: Mutation prediction for the deep-sequencing age. Nat. Methods.

[43] Rentzsch P., Witten D., Cooper G.M., Shendure J., Kircher M. (2019). CADD: Predicting the deleteriousness of variants throughout the human genome. Nucleic Acids Res..

[44] Yeo G., Burge C.B. (2004). Maximum entropy modeling of short sequence motifs with applications to RNA splicing signals. J. Comput. Biol..

[45] Jumper J., Evans R., Pritzel A., Green T., Figurnov M., Ronneberger O., Tunyasuvunakool K., Bates R., Žídek A., Potapenko A. (2021). Highly accurate protein structure prediction with AlphaFold. Nature.

