# Supplementary material for: Association Study Between NOTCH2 Gene and Idiopathic Central Precocious Puberty in Korean Girls
Source: Int J Mol Sci. 2026 Jul 27;27(15):6686. doi: 10.3390/ijms27156686 (PMC13467111; doi:10.3390/ijms27156686)
Supplement: Supplementary file 1 [file ijms-27-06686-s001.zip › ijms-4362314-supplementary.pdf]

Supplementary Table S1. Primer sequences, amplicon sizes, and annealing temperatures for Sanger sequencing of all 34 coding exons of *NOTCH2* (NM_024408.6)

Amplicon	Target Exon	Forward Primer (5'→3')	Reverse Primer (5'→3')	Product Size (bp)	Annealing Temp (°C)
NOTCH2-1	Ex1	CAC ACG AGG CTG CTT CGT	CGG CGA TGT CCA AAC TCT T	493	60
NOTCH2-2	Ex2	GGA TGG GCT TCT GTG CTA GA	CCA GGA AAG CTC AAG AAC CAG	363	60
NOTCH2-3	Ex3	AAA GGA CAT GGG AGT ATG TGG T	TGT TAA ATG TGC ACC AGA GC	572	62
NOTCH2-4	Ex4	TGC TGA GGT CCT TGG AGA GT	TCA CTT CCC TTT TTC CTT GG	641	60
NOTCH2-5	Ex5	TAT GGT GAG TCC TGG GTG AG	GCA TGG AGA TCC TGC TCA GT	399	60
NOTCH2-6	Ex6	CTG GAG CTC TGC TTG CCT TA	TTT CTG GAA CAG TCC TGA GTG A	493	60
NOTCH2-7	Ex7	CAA GCA TGT CCC CAA GGT	ACA CTG GCC ATG TAG CAA GA	459	60
NOTCH2-8	Ex8	CTG GCC CTG ATG CTA TAA CC	GCT TGT GAA CCC TGA CTT CC	443	60
NOTCH2-9	Ex9	TCT CAT CAT GGC AGA GTT CCT	CAC ACT CCT ATC ACC AAG CAC T	393	60
NOTCH2-10	Ex10	AAG TGC CTC TGA TTC CCT GA	CAG CTC TTC CTA AAC ACA GAG GA	426	60
NOTCH2-11	Ex11	CCT GCA GTA TTA ATT GAC CAC A	GCT TTG GCT GTG CTT CTC AGa	489	60
NOTCH2-12	Ex12	TGA ACG AGT CTG TGG ACT GC	CTA ACC CTC CAG AGT GAC AAA G	390	60
NOTCH2-13	Ex13	TTA TGG CCT GAG CAG ATG AA	GCC CCA TTT GAC AAC TTC AG	392	60
NOTCH2-14	Ex14	TGC AAG GTG ATG CAA GGA TA	TCC CCA AGT GCC TTA CAA GT	434	60
NOTCH2-15	Ex15	—b	GCA GGA AGG ACA ACT GAG GA	374	60
NOTCH2-16	Ex16	AAG ATC ATG GGC TTC TCC ATT	GCT CCT TTG CAA AAC CTG TG	400	61
NOTCH2-17	Ex17	GGG CCA ACC AGT TTG TCT GT	CTG GAT AAA TGA CCG GTA TGC	396	60
NOTCH2-18	Ex18	GAG GGA GTT GGC AAA ATT GA	GAC TGT GGA CTG GGA TCC AT	496	60
NOTCH2-19	Ex19	CTT CTG GCA CCC AAT CTA GC	CCA GCA CCA TTT TGG AAT CT	532	60
NOTCH2-20	Ex20	TTG TCC TTA AAG TCA CAG TTT CCA	—c	—c	60
NOTCH2-21	Ex21	GCA TCT GGT ACT GAG GGT TG	GGT AAA CAT TAT GAC GGG GAT T	471	60
NOTCH2-22	Ex22	CAG GTT TTC TGT CCC TGC AT	TGG GAG AAT AAA ATG GCT TGAd	476	58
NOTCH2-23	Ex23	CAT GCG GAG CCC TAG TAT GT	AAA ATT AAG CTG CTG TTG TTT TTC	593	60
NOTCH2-24	Ex24	ACA GGC CAA TCA TGT TGA GA	AAA CAG CTT TAA ATG GAA AAA CC	462	60
NOTCH2-25	Ex25	CTA TGC CTC GGG AGA TAC CA	CCA TTG TCA GCA AGG GAG AG	843	60
NOTCH2-26	Ex26	AGA AGG TTT TGT GGG GCA TT	TCT TAA GTG GTG GGC CAT TC	620	60
NOTCH2-27	Ex27	CTT AAG GCA GCA TCC CAG AG	GGG ATT CAC CTG AAA GTC CA	489	60
NOTCH2-28	Ex28	TTG GGG GAA CAA TTT TCA TT	TTC AGT CAC TCA CAA GGC ATT T	498	58
NOTCH2-29	Ex29	GAT GAA TGC CAA GGG AAT GT	TTG TCT GGG TAA GAC ATC AAG A	392	59
NOTCH2-30	Ex30	GAG GGG TGG AGG AGT AGG AA	GCA TTC ATT TCC TCG AGC TG	438	62
NOTCH2-31	Ex31	GGT GGC AAA TGT GAA ACT AAT AA	CCT GCC CTA ATC TCT CAA ATG	574	60
NOTCH2-32	Ex32	TTG GGC ACA AAG ATA ACA AGA	TGA ATG AAA GAA TGA GTG AAT CTC	423	60
NOTCH2-33	Ex33	TGG TTT TTC CTA TAG ACC CCT ACA	GCT CTC GAA ACC CAG TGT CT	377	60
NOTCH2-34-1	Ex34 (part 1)	TTC AGT ACA TTG GTA GAG GCA CA	CCA GGG GAT GTA ATC ATT GG	659	60
NOTCH2-34-2	Ex34 (part 2)	GCC CAA CAG ATC TTT CCT CA	GAG GGG TGG TTA TGT GCT TC	—e	60
NOTCH2-34-3	Ex34 (part 3)	CTT CCC TCA GTG AGC CAG TT	TGA CCA CTG TGA CTG GGT GT	569	60
NOTCH2-34-4	Ex34 (part 4)	CTG CCC ACC ATG TAC CAG AT	TGA CGA ATT GCT TCT CTT GC	576	60

Abbreviation: Ex, exon; bp, base pairs; Annealing Temp, annealing temperature used in the PCR thermocycling program. All primer sequences are listed in the 5'→3' direction. Genomic reference: *NOTCH2* NM_024408.6; chromosomal coordinates based on GRCh38/hg38 (chromosome 1).

a: For amplicon NOTCH2-11, an alternative internal reverse primer (NOTCH2-11R1: 5′-TGA CTT CTC CAC TGG CTA GG-3′; 20-mer, Tm 62°C) was used for independent confirmation of identified variants in this amplicon, yielding a product of 539 bp.

b: The forward primer sequence for amplicon NOTCH2-15 (NOTCH2-15F) was not available from the original laboratory protocol record at the time of manuscript preparation. Sequencing of this region was confirmed using the reverse primer (NOTCH2-15R) in conjunction with the reverse primer of the adjacent NOTCH2-14 amplicon, and complete coverage of exon 15 was verified by sequence alignment. The original primer sequence is available upon request from the corresponding author.

c: The reverse primer sequence and expected product size for amplicon NOTCH2-20 (NOTCH2-20R) were not available from the original laboratory record. Successful amplification and sequencing of exon 20 were confirmed by sequence alignment to the reference (NM_024408.6). Details are available from the corresponding author upon request.

d: For amplicon NOTCH2-22, sequencing analysis was performed using only the reverse primer (NOTCH2-22R) owing to suboptimal sequence quality from the forward primer in this GC-rich region.

e: The amplicon size for NOTCH2-34-2 was not documented in the original laboratory record. Sequence continuity between NOTCH2-34-1 and NOTCH2-34-3 was confirmed by alignment, ensuring complete coverage of exon 34.

Supplementary Table S2. Complete catalogue of non-coding and synonymous variants identified in the *NOTCH2* gene

No.	Exon	HGVS (cDNA)	Protein Change	dbSNP	ClinVar	MaxEntScan Delta	Control n/200 (%)	HWE *P* (Control)	CPP Patient n/200 (%)	HWE *P* (CPP patient)	Uncorrected *P*-value	Corrected *P*-value
Synonymous variants												
1	Exon 1	c.15C>T	p.Arg5Arg	rs4021006	B	N/A	8/200 (4.0%)	>0.05	10/200 (5.0%)	>0.05	0.660	1.000
2	Exon 1	c.45C>T	p.Leu15Leu	rs782485593	LB	N/A	0/200 (0.0%)	N/A (AF=0)	1/200 (0.5%)	N/A (singleton)	1.000	1.000
3	Exon 10	c.1653G>A	p.Pro551Pro	rs187731832	LB	N/A	0/200 (0.0%)	N/A (AF=0)	1/200 (0.5%)	N/A (singleton)	1.000	1.000
4	Exon 19	c.3034T>C	p.Leu1012Leu†	rs77194332	B	N/A	5/200 (2.5%)	>0.05	5/200 (2.5%)	>0.05	1.000	1.000
5	Exon 25	c.4014C>T	p.Ser1338Ser	rs17024525	B	N/A	29/200 (14.5%)	>0.05	29/200 (14.5%)	>0.05	1.000	1.000
6	Exon 25	c.4305G>A	p.Arg1435Arg	rs6692009	LB	N/A	0/200 (0.0%)	N/A (AF=0)	1/200 (0.5%)	N/A (singleton)	1.000	1.000
7	Exon 30	c.5454G>C	p.Val1818Val	Novel	NE	-0.3	1/200 (0.5%)	N/A (singleton)	0/200 (0.0%)	N/A (AF=0)	1.000	1.000
8	Exon 34	c.6045T>C	p.Phe2015Phe	rs766885963	LB	N/A	0/200 (0.0%)	N/A (AF=0)	1/200 (0.5%)	N/A (singleton)	1.000	1.000
9	Exon 34	c.6150T>A	p.Ala2050Ala	rs764208739	LB	N/A	0/200 (0.0%)	N/A (AF=0)	1/200 (0.5%)	N/A (singleton)	1.000	1.000
10	Exon 34	c.6222C>T	p.Thr2074Thr	rs764335447	LB	N/A	1/200 (0.5%)	N/A (singleton)	0/200 (0.0%)	N/A (AF=0)	1.000	1.000
11	Exon 34	c.6421C>T	p.Leu2141Leu	rs3795666	B	N/A	8/200 (4.0%)	>0.05	9/200 (4.5%)	>0.05	1.000	1.000
12	Exon 34	c.7263T>A	p.Ser2421Ser	rs369912969	LB	N/A	0/200 (0.0%)	N/A (AF=0)	1/200 (0.5%)	N/A (singleton)	1.000	1.000
13	Exon 34	c.7341T>A	p.Gly2447Gly	rs6685892	B	N/A	6/200 (3.0%)	>0.05	3/200 (1.5%)	>0.05	0.510	1.000
Missense variants												

1	Exon 4	c.703A>T	p.Thr235Ser †	rs200464440	LB	N/A (coding)	0/200 (0%)	N/A (AF=0)	1/200 (0.5%)	N/A (singleton)	1.000	1.000
2	Exon 4	c.710G>A	p.Arg237Gln ‡	rs146498360	VUS	N/A (coding)	2/200 (1.0%)	>0.05	0/200 (0%)	N/A (AF=0)	0.498	1.000
3	Exon 13	c.2042T>A	p.Ile681Asn	rs74882029	LB	N/A (coding)	1/200 (0.5%)	N/A (singleton)	1/200 (0.5%)	N/A (singleton)	1.000	1.000
4	Exon 18	c.2816C>T	p.Pro939Leu	rs201100122	VUS	N/A (coding)	1/200 (0.5%)	N/A (singleton)	1/200 (0.5%)	N/A (singleton)	1.000	1.000
5	Exon 23	c.3779G>A	p.Arg1260His	rs75423398	LB	N/A (coding)	12/200 (6.0%)	>0.05	6/200 (3.0%)	>0.05	0.180	1.000
6	Exon 25	c.4081G>A	p.Ala1361Thr †	rs138567855	VUS	N/A (coding)	0/200 (0%)	N/A (AF=0)	1/200 (0.5%)	N/A (singleton)	1.000	1.000
7	Exon 28	c.5065A>T	p.Ile1689Phe ‡*	rs60854092	VUS	N/A (coding)	13/200 (6.5%)	0.680	0/200 (0%)	N/A (AF=0)	0.0002*	0.011*
8	Exon 30	c.5357G>A	p.Arg1786Gln ‡	rs587634422	VUS	N/A (coding)	1/200 (0.5%)	N/A (singleton)	0/200 (0%)	N/A (AF=0)	1.000	1.000
9	Exon 33	c.5975C>T	p.Ala1992Val †§	Novel§	NE	N/A (coding)	0/200 (0%)	N/A (AF=0)	1/200 (0.5%)	N/A (singleton)	1.000	1.000
10	Exon 33	c.6008G>A	p.Arg2003Gln ‡	rs142978073	LB	N/A (coding)	1/200 (0.5%)	N/A (singleton)	0/200 (0%)	N/A (AF=0)	1.000	1.000
11	Exon 34	c.6892C>T	p.Arg2298Trp	rs1270274564	VUS	N/A (coding)	1/200 (0.5%)	N/A (singleton)	1/200 (0.5%)	N/A (singleton)	1.000	1.000
Intronic Variants												
1	Intron 4 (IVS4)	T>G		rs2453040		0.00	6/200 (3.0%)	>0.05	3/200 (1.5%)	>0.05	0.510	1.000
2	Intron 5 (IVS5)	A>G		rs782732252		0.00	1/200 (0.5%)	N/A (singleton)	0/200 (0.0%)	N/A (AF=0)	1.000	1.000
3	Intron 5 (IVS5)	A>G		rs2493409		0.00	200/200 (100.0%)‡	N/A (monomorphic)	200/200 (100.0%)‡	N/A (monomorphic)	N/A	N/A
4	Intron 6 (IVS6)	T insertion		rs587608977		0.00	1/200 (0.5%)	N/A (singleton)	3/200 (1.5%)	>0.05	0.624	1.000
5	Intron 8 (IVS8)	G>T		rs183831820		0.00	1/200 (0.5%)	N/A (singleton)	2/200 (1.0%)	>0.05	1.000	1.000
6	Intron 8	G>A		Novel		0.00	1/200	N/A	0/200	N/A (AF=0)	1.000	1.000

	(IVS8)						(0.5%)	(singleton)	(0.0%)			
7	Intron 10 (IVS10)	C>T		rs2934381		0.00	6/200 (3.0%)	>0.05	3/200 (1.5%)	>0.05	0.510	1.000
8	Intron 13 (IVS13)	T>G		rs7534586		0.00	6/200 (3.0%)	>0.05	3/200 (1.5%)	>0.05	0.510	1.000
9	Intron 13 (IVS13)	T>A		rs7534585		0.00	6/200 (3.0%)	>0.05	3/200 (1.5%)	>0.05	0.510	1.000
10	Intron 14 (IVS14)	G>T		rs1387059585		0.00	0/200 (0.0%)	N/A (AF=0)	1/200 (0.5%)	N/A (singleton)	1.000	1.000
11	Intron 14 (IVS14)	T>C		rs782298963		0.00	0/200 (0.0%)	N/A (AF=0)	1/200 (0.5%)	N/A (singleton)	1.000	1.000
12	Intron 15 (IVS15)	C>A		rs1470443129		0.00	0/200 (0.0%)	N/A (AF=0)	1/200 (0.5%)	N/A (singleton)	1.000	1.000
13	Intron 16 (IVS16)	C>A		rs149097658		0.00	4/200 (2.0%)	>0.05	4/200 (2.0%)	>0.05	1.000	1.000
14	Intron 17 (IVS17)	C>T		Novel		0.00	14/200 (7.0%)	>0.05	10/200 (5.0%)	>0.05	0.430	1.000
15	Intron 19 (IVS19)	G>A		Novel		0.00	6/200 (3.0%)	>0.05	3/200 (1.5%)	>0.05	0.510	1.000
16	Intron 22 (IVS22)	G>A		Novel		0.00	6/200 (3.0%)	>0.05	3/200 (1.5%)	>0.05	0.510	1.000
17	Intron 24 (IVS24)	T>G		Novel		0.00	0/200 (0.0%)	N/A (AF=0)	1/200 (0.5%)	N/A (singleton)	1.000	1.000
18	Intron 24	A>G		Novel		0.00	0/200 (0.0%)	N/A (AF=0)	1/200 (0.5%)	N/A (singleton)	1.000	1.000

	(IVS24)											
19	Intron 28 (IVS28)	C>T		Novel		0.00	1/200 (0.5%)	N/A (singleton)	0/200 (0.0%)	N/A (AF=0)	1.000	1.000
20	Intron 29 (IVS29)	G>A		Novel		0.00	1/200 (0.5%)	N/A (singleton)	0/200 (0.0%)	N/A (AF=0)	1.000	1.000
21	Intron 30 (IVS30)	T>C		Novel		0.00	0/200 (0.0%)	N/A (AF=0)	2/200 (1.0%)	>0.05	0.498	1.000
INDEL Variant												
1	Intron 6 / Exon 7 boundary	T insertion; poly-T repeat expansion (TTTT→ TTTTT, heterozygous)		Novel			1/200 (0.5%)	N/A (singleton)	3/200 (1.5%)	>0.05	0.624	1.000
5′UTR variants (SNVs)												
1	Ex1	c.−14A>C		Novel			2/200 (1.0%)	>0.05	6/200 (3.0%)	>0.05	0.282	1.000
2	Ex1	c.−21G>A		Novel			1/200 (0.5%)	N/A (singleton)	0/200 (0.0%)	N/A (AF=0)	1.000	1.000
3	Ex1	c.−23C>A		Novel			0/200 (0.0%)	N/A (AF=0)	1/200 (0.5%)	N/A (singleton)	1.000	1.000
4	Ex1	c.−97G>C		Novel			1/200 (0.5%)	N/A (singleton)	2/200 (1.0%)	>0.05	1.000	1.000
5	Ex1	c.−133C>T		Novel			1/200 (0.5%)	N/A (singleton)	0/200 (0.0%)	N/A (AF=0)	1.000	1.000
6	Ex1	c.−169G>T		Novel			7/200 (3.5%)	>0.05	5/200 (2.5%)	>0.05	0.796	1.000
7	Ex1	c.−236C>G		Novel			0/200	N/A (AF=0)	1/200	N/A	1.000	1.000

							(0.0%)		(0.5%)	(singleton)		
5′UTR variants (INDELs)												
8	Ex1	c.−40_−38dup (GGC trinucleotide repeat expansion; 6 → 7 copies) §	INDEL (insertion)	Novel			2/200 (1.0%)	>0.05	4/200 (2.0%)	>0.05	0.681	1.000
9	Ex1	c.−204_−179 del (26 bp deletion)	INDEL (deletion)	Novel			0/200 (0.0%)	N/A (AF=0)	1/200 (0.5%)	N/A (singleton)	1.000	1.000

No variant showed significant departure from Hardy-Weinberg equilibrium (HWE) in either group (all $P > 0.05$).

HWE P values were assessed by chi-square goodness-of-fit test separately in CPP and control groups; N/A(AF=0) = trivially satisfied (allele frequency = 0); N/A(sgl) = singleton, excluded from HWE testing; all non-singleton variants satisfied HWE (P greater than 0.05).

All variants were identified by direct Sanger sequencing of the full coding and flanking intronic regions of NOTCH2 (transcript reference NM_024408.6; chromosome 1, GRCh38/hg38) in 100 patients with central precocious puberty (CPP) and 100 healthy controls. Allele frequencies were calculated over 200 alleles per group. Statistical significance was assessed by two-tailed Fisher's exact test; no variant in this supplementary table reached the threshold of $P < 0.05$. Chromosomal positions are provided on the minus strand orientation of chromosome 1.

MaxEntScan delta scores were calculated using the maximum entropy model for all intronic variants [44]. For 5' splice donor sites, the canonical scoring window encompasses positions −3 to +6 relative to the exon-intron junction (9-mer); for 3' splice acceptor sites, the window encompasses positions −20 to +3 (23-mer). All 21 intronic variants were located outside these canonical scoring windows (minimum distance: 24 bp from the nearest splice site, intronic variant No. 14 [Intron 17]; maximum distance: approximately 1,900 bp). Accordingly, all MaxEntScan delta scores were 0.00, indicating no disruption of canonical splice site motifs. This finding is consistent with all SpliceAI component scores (DG, DL, AG, AL) remaining below the 0.2 significance threshold [30].

† p.Leu1012Leu (rs77194332) is located within the cbEGF-like repeat domain of NOTCH2 (residue 1012 falls outside the ANK repeat region, which spans residues ~1,851–2,126), which harbours functionally significant missense variants in the NOTCH2 receptor; the synonymous nature of this variant precludes direct functional inference.

‡ rs2493409 (Intron 5): All 100 subjects in both groups were confirmed homozygous for the G allele. The allele frequency of 100% in both groups reflects a population-fixed alternate allele relative to the GRCh38 reference in the Korean study population. This variant is monomorphic in this cohort and was retained in the catalogue for completeness; it was excluded from all association analyses.

§ The GGC insertion at No. 8 is located within a GGC trinucleotide repeat tract immediately upstream of the NOTCH2 5′UTR. The variant represents an expansion from 6 to 7 repeat copies. The precise anchor position (chr1:120,069,444 or 120,069,446) is ambiguous owing to the repetitive context; the duplication is annotated according to HGVS 3′-shift rules as c.−40_−38dup. The potential impact on NOTCH2 transcriptional regulation has not been functionally assessed.

This INDEL is located immediately adjacent to the intronic T insertion recorded as rs587608977 (Part B, No. 4) within a poly-T repeat tract at the Intron 6/Exon 7 boundary. The two events likely represent distinct sequencing observations within the same low-complexity region. Neither variant reached statistical significance.
HGVS: Human Genome Variation Society nomenclature; CPP: central precocious puberty; HWE: Hardy-Weinberg equilibrium, IVS: intervening sequence (intron); dbSNP: Single Nucleotide Polymorphism database (NCBI); Novel: not previously registered in dbSNP at the time of analysis; —: chromosomal position not captured in the primary dataset (HGVS cDNA notation provided as primary locator).
ClinVar classification: B = Benign; LB = Likely Benign; VUS = Variant of Uncertain Significance; NE = Not Entered.
All allele frequencies were calculated from 200 alleles per group (100 diploid subjects). For heterozygous carriers, one allele was counted; no homozygous alternate genotypes were observed among the variants in this study. *P*-values were derived by two-tailed Fisher's exact test. No Bonferroni correction was applied to the supplementary variants; the Bonferroni-corrected threshold for 55 total variants tested is $P < 0.000909$. Transcript reference: NM_024408.6 (NOTCH2, Homo sapiens).
